# The mutational and clinical spectrum of *EYS*-associated retinitis pigmentosa in an Eastern European and Northern Asian cohort: two recurrent founder alleles

**DOI:** 10.3389/fgene.2026.1951476

**Published:** 2026-09-28

**Authors:** Marianna Weener, Igor Hatsenko, Inna Zolnikova, Natella Chincharauli, Maria Shilova, Anna Venger, Olga Luneva, Larisa Balashova, Jean Salmasi, Gennadiy Poryadin

**Affiliations:** 1 Ocular Genomics Institute, Massachusetts Eye and Ear, Harvard Medical School, Boston, MA, United States; 2 Oftalmic Clinical Research Center, Moscow, Russia; 3 Morozovskaya Children’s City Clinical Hospital, Moscow, Russia; 4 Helmholtz National Medical Research Center of Eye Diseases, Moscow, Russia; 5 I.M. Sechenov First Moscow State Medical University, Moscow, Russia; 6 Ophthalmology Clinic of Dr. Shilova, Moscow, Russia; 7 EYS Patients Advocacy Group, Moscow, Russia; 8 Pirogov Russian National Research Medical University, Moscow, Russia

**Keywords:** *EYS*, founder mutation, genotype-phenotype correlation, inherited retinal disease, retinitis pigmentosa, structural variants, whole-exome sequencing

## Abstract

**Purpose:**

Biallelic *EYS* variants are one of the frequent causes of autosomal recessive inherited retinal disease worldwide, yet the *EYS* mutational landscape of Eastern European and Northern Asian populations has not been systematically described. We characterized the molecular and clinical spectrum of *EYS*-associated retinitis pigmentosa (RP) in this population.

**Methods:**

Thirty-nine patients with confirmed biallelic *EYS* variants were selected from 500 patients enrolled in the prospective inherited retinal disease genotyping clinical trial (NCT03901391). All patients underwent whole exome sequencing with copy number/structural variant analysis and standardized ophthalmic phenotyping, including best corrected visual acuity (BCVA), slit lamp biomicroscopy, refractometry, perimetry, optical coherent tomography (OCT), fundus autofluorescence (FAF), ophthalmoscopy and electroretinogram (ERG).

**Results:**

We identified 34 distinct *EYS* variants: 22 pathogenic, 8 likely pathogenic, and 4 variants of uncertain significance by American College of Medical Genetics (ACMG) criteria. Fifteen variants were novel, including eight structural deletions. Two recurrent alleles c.1155T>A (p.Cys385*) in 16 (20.3%) alleles, and c.8648_8655del (p.Thr2883Lysfs*4) in 15 (19.0%) alleles were the most frequent; together they accounted for 39.2% of alleles, with relatedness analysis supporting a shared founder origin. Median age of disease manifestation was 18 years and median BCVA 0.16 logMAR; worse acuity correlated with older age (rank correlation coefficient = 0.34, p = 0.035).

**Conclusion:**

This first description of the *EYS* spectrum in an Eastern European and Northern Asian cohort expands the allelic diversity of the gene, provides a genotype-phenotype description and defines two population-specific founder alleles amenable to targeted screening and future treatment.

## 1 Introduction

Retinitis pigmentosa (RP) is the most common inherited retinal disease (IRD), affecting roughly 1 in 4,000 individuals and characterized by progressive rod-then-cone photoreceptor degeneration that manifests as nyctalopia, concentric visual field constriction, and eventual loss of central vision. Autosomal recessive RP (arRP) is genetically heterogeneous, with 79 causative genes. Among these, the *EYS* gene (Eyes shut homolog; RP25, OMIM 612424) is one of the most frequent causes of arRP (Abd El-Aziz et al., 2008; Collin et al., 2008; Verbakel et al., 2018).

*EYS* gene is located on chromosome 6q12, spans almost 2 Mb of genomic DNA and comprises 43 exons, reference transcript is NM_001142800.2 (ENST00000503581). It encodes a large secreted extracellular matrix protein expressed in the photoreceptor outer segments, where it is required for the maintenance of ciliary architecture and photoreceptor survival (Abd El-Aziz et al., 2008; Collin et al., 2008). Biallelic loss-of-function (LoF) variants in *EYS* cause photoreceptor disorganization and apoptosis, producing a progressive rod–cone dystrophy (Marques et al., 2025).

The reported contribution of *EYS* to arRP varies markedly by population, from approximately 5% in European and North-American cohorts to 18%–33% in East-Asian, and especially Japanese populations, where specific recurrent alleles reflect founder effects (Littink et al., 2010; Sun et al., 2020; Gao et al., 2022; Yang et al., 2020; Arai et al., 2015; Iwanami et al., 2019; Koyanagi et al., 2019; Barragán et al., 2010). Because *EYS* is large and structurally complex, its variant spectrum is enriched for copy number and structural variants (SVs), which are frequently missed by panel or exome sequencing that is not complemented by dedicated SV analysis. Characterizing the population-specific allelic frequency of *EYS* gene is relevant to diagnostic strategy and to the design of allele- and mutation-directed therapies (Iwanami et al., 2019; Zampaglione et al., 2020).

Population size of Eastern Europe and Northern Asia is more than 200 million people, and ethnic diversity of the populations encompass more than 190 recognized ethnic groups across the Russian Federation and neighboring states. Mutational spectrum of cone dystrophy and achromatopsia in this region was reported earlier (Ivanova et al., 2020; Zolnikova et al., 2017), but the rod-cone dystrophy mutational spectrum in this region has not been systematically evaluated. We therefore set out to define the molecular and clinical spectrum of *EYS*-associated RP in a cohort of 39 patients with confirmed molecular diagnosis, derived from a prospective genotyping study of 500 patients with RP (NCT03901391).

## 2 Materials and methods

### 2.1 Study design, participants, and ethics

This study was conducted within a prospective observational inherited retinal disorders (IRD) genotyping clinical trial (NCT03901391) carried out at the Oftalmic Clinical Research Center, Moscow. The study was approved by the Ethics Committee at UDP CKB, Moscow (approval number IRB#007, 26 June 2019) and adhered to the tenets of the Declaration of Helsinki. All participants provided written informed consent. 361/500 cases (72.2%) were molecularly solved and 39/361 (10.8%) patients were confirmed carrying biallelic *EYS* variants as a cause of RP diagnosis and were included in the analyses reported here (n = 39).

### 2.2 Clinical phenotyping

All participants underwent standardized ophthalmology examination including best corrected visual acuity (BCVA), slit lamp biomicroscopy, refractometry, intraocular pressure measurement, dilated fundus examination, automated perimetry, ERG, fundus autofluorescence, and optical coherence tomography (OCT). BCVA was measured monocularly (OD and OS) using a standard illuminated chart and recorded in decimal notation; for analysis, decimal acuities were converted to logMAR (logMAR = −log_10_[decimal]) and the mean of the two eyes was used. Objective refraction was obtained by autorefractometry; the spherical equivalent (SE) of each eye was calculated and averaged across eyes to yield a single representative value per participant. Automated perimetry (Humphrey Field Analyzer, Carl Zeiss Meditec) was used to estimate the size and shape of the visual field.

SD-OCT was performed under standardized conditions for high resolution macular imaging (Zeiss, CIRRUS 6000). Fundus imaging was performed using Topcon TRC-NW8 fundus camera. Ganzfeld (full-field) ERG was recorded according to the standards of the International Society for Clinical Electrophysiology of Vision (ISCEV). For each subject, a- and b-wave amplitudes and latency times were analyzed separately and averaged. Quantitative ERG was available for all 39 patients (36 with recordable responses and 3 non-recordable). Ultrawide-field (Optos) imaging was not available at the study site and was therefore not obtained.

### 2.3 DNA extraction, sequencing, and variant calling

Genomic DNA was extracted from peripheral blood using a Qiagen DNA kit. Whole exome sequencing was performed on an Illumina NovaSeq 6000 platform (2 × 100 bp) with target enrichment using the Agilent SureSelect Human All Exon V7 panel, to a mean on-target depth of 70–100×. Reads were aligned and variants called using an automated pipeline against the human reference genome hg38. The method did not reliably detect insertions/deletions longer than 10 bp or intronic variants (other than those adjacent to exons); such variants were addressed by dedicated copy number/structural variant analysis and multiplex ligation-dependent probe amplification (MLPA).

### 2.4 Structural variant detection and confirmation

Copy number and structural variants in *EYS* were identified from exome coverage data and confirmed by MLPA using a standardized protocol. Structural deletion breakpoints are reported in genomic coordinates hg38 (Table 1; Supplementary Table 1).

**TABLE 1:** The 34 distinct *EYS* variants identified in the cohort**.** Variants are ordered along the coding sequence (NM_001142800.2).

Nucleotide change (NM_001142800.2)	Protein change	Location	Variant type	N	gnomAD v4.1 MAF	ACMG class	References
c.32dupT	p.Met12Aspfs*14	Exon 4	Frameshift	2	1.55 × 10^−5^	LP	Sun et al. (2020)
c.105delA	p.Gln34fs	Exon 2	Deletion	1	1.24 × 10^−6^	P	–
c.525_527delGGA	p.Glu176del	Exon 4	Frameshift	1	5.95 × 10^−5^	VUS	Barragán et al. (2010)
c.600C>A	p.Cys200*	Exon 4	Nonsense	1	–	P	Novel
c.1057-1G>C	–	Intron 6	Splicing	2	–	P	Barragán et al. (2010)
c.1155T>A	p.Cys385*	Exon 7	Nonsense	16	2.98 × 10^−4^	LP	Yang et al. (2020)
c.1641_1644delTCAG	p.Ser547fs	Exon 11	Frameshift	1	–	P	Novel
c.1956C>A	p.Cys652*	Exon 12	Nonsense	2	–	P	Novel
c.2259+1G>A	–	Intron 14	Splicing	4	5.83 × 10^−5^	P	Yang et al. (2020)
c.2829T>A	p.Cys943*	Exon 18	Nonsense	1	–	P	Westin et al. (2021)
c.2989_2990insAA	–	Intron 19	Splicing	1	–	P	Novel
c.3676G>T	p.Gly1226*	Exon 24	Nonsense	4	6.7 × 10^−7^	LP	Woof et al. (2024)
c.4120C>T	p.Arg1374*	Exon 26	Nonsense	2	1.09 × 10^−5^	P	Woof et al. (2024)
c.4350_4356del	p.Ile1451Profs*3	Exon 26	Frameshift	1	9.47 × 10^−5^	LP	Marques et al. (2025)
c.4815_4816delCAinsATAG	p.Thr1606fs	Exon 26	Frameshift	2	–	P	Novel
c.5084C>G	p.Ser1695*	Exon 26	Nonsense	2	6.45 × 10^−7^	P	Woof et al. (2024)
c.5835+1G>A	–	Intron 27	Splicing	1	7.12 × 10^−7^	P	Heutinck et al. (2025)
c.6714del	p.Ile2239Serfs*18	Exon 33	Frameshift	4	5.22 × 10^−5^	LP	Westin et al. (2021)
c.7056-1G>A	–	Intron 35	Splicing	1	–	P	Novel
c.7276T>G	p.Tyr2426Asp	Exon 37	Missense	1	4.51 × 10^−6^	VUS	–
c.7394C>G	p.Thr2465Ser	Exon 37	Missense	1	3.54 × 10^−4^	VUS	Gao et al. (2022)
c.7550G>A	p.Gly2517Asp	Exon 38	Missense	1	–	VUS	Novel
c.8133_8137delCTTTC	p.Phe2712fs	Exon 42	Frameshift	2	3.67 × 10^−5^	LP	Di et al. (2016)
c.8648_8655del​CAT​GCA​GA	p.Thr2883Lysfs*4	Exon 43	Frameshift	15	3.65 × 10^−4^	LP	Di et al. (2016)
c.8831dup	p.Arg2946LysfsTer4	Exon 43	Frameshift	1	–	P	Woof et al. (2024)
c.9317_9336del	p.Thr3106LysfsTer13	Exon 43	Frameshift	1	4.52 × 10^−5^	LP	Westin et al. (2021)
chr6:63984236-63984906	671bp deletion	Exon 35	Deletion	1	–	P	Novel
chr6:65291239-65294449	3 kb deletion	Intron 12	Deletion	1	–	P	Novel
chr6:65297039-65131851	165 kb deletion	Exon 11	Deletion	1	–	P	Novel
chr6:65490945-65498976	8.1 kb deletion	Exon 3-4	Deletion	1	–	P	Novel
chr6:65491560-65384458	107 kb deletion	Exon 4-7	Deletion	1	–	P	Novel
chr6:65497059-65352499	145.4 kb deletion	Exon 6-8	Deletion	1	–	P	Novel
chr6:65504108-65515495	11.1 kb deletion	Intron 2	Deletion	1	–	P	Novel
chr6:65508505-65382463	126.7 kb deletion	Exon 5-7	Deletion	1	–	P	Novel

N = allele count in the cohort. MAF = minor allele frequency, gnomAD v4.1. ACMG classification of P, pathogenic; LP, likely pathogenic; VUS, variant of uncertain significance. The two founder alleles, c.1155T>A (p.Cys385*) and c.8648_8655del (p.Thr2883Lysfs*4), are the most frequent. Structural deletions are given as approximate hg38 coordinates with size.

### 2.5 Variant classification (ACMG/AMP)

Sequence variants were classified according to the American College of Medical Genetics (ACMG) and the Association for Molecular Pathology (AMP) standards and guidelines (Richards et al., 2015). Each variant was assigned a tier of pathogenic (P), likely pathogenic (LP), or variant of uncertain significance (VUS) and the specific ACMG/AMP criteria applied to each variant are listed in Supplementary Table 1. For previously reported variants, ClinVar and published classifications were considered as supporting evidence; for novel variants (which by definition have no ClinVar entry), classification was performed *de novo* from the criteria, and the basis of classification is recorded in a separate column.

### 2.6 Determination of variant novelty

A variant was considered novel if it was absent from gnomAD v4.1, ClinVar, the Human Gene Mutation Database (HGMD), and the peer-reviewed *EYS* literature at the time of analysis. Structural variants were additionally checked for absence from population structural variant resources: gnomAD-SV and Database of Genomic Variants (DGV). Population allele frequencies are reported from gnomAD v4.1 (Table 1; Supplementary Table 1).

### 2.7 Ethnicity and genetic ancestry

Ethnicity was first self-reported by participants on the basis of passport data and is provided to support future population-genetic and founder-mutation research in specific communities; we note that ethnicity is a fluid socio-political construct and that no single internationally recognised classification exists, so labels follow common regional usage and are standardised *post hoc* in line with Ethnonational Classification demographic recommendations (Ahmed et al., 2004). Self-reported ethnicity was then compared with genetically inferred ancestry from the whole exome sequencing (WES) data: individual germline variant profiles were projected against 1000 Genomes Project reference populations EUR, EAS, SAS, AFR, AMR–European, east Asian, south Asian, African and American respectively, and individual ancestry proportions were estimated by maximum-likelihood admixture analysis (ADMIXTURE and fastSTRUCTURE). We report self-reported ethnicity and genetically inferred ancestry as distinct categories and do not treat heterogeneous ethnic labels as equivalent taxonomic units in any analysis (Ahmed et al., 2004; Gusareva et al., 2025; Alexander et al., 2009; Raj et al., 2014).

### 2.8 Relatedness analysis

Because recurrent alleles suggest a possible founder effect, the exomes were analyzed for relatedness. Following joint calling through a single consistent pipeline (to reduce exome capture bias) and quality control, a filtered set of high quality SNPs (single nucleotide polymorphisms) in KING software version 2.3.2 on a filtered, high quality SNP set (12,300 SNPs per sample) on the hg38 build was used to estimate pairwise kinship (Manichaikul et al., 2010). Kinship coefficients were interpreted using standard KING thresholds (first-degree 0.177–0.354; second-degree 0.0884–0.177; third-degree 0.0442–0.0884; <0.0442 unrelated/more distant). The analyzed population was not considered small or endogamous, so background relatedness is not expected to inflate kinship estimates. The mean ± SD coefficient was 0.0092 ± 0.0034 and range of pairwise kinship coefficients among the founder allele carriers was from 0.0012 to 0.0531.

### 2.9 Segregation analysis

Segregation of all detected *EYS* variants was confirmed by PCR (polymerase chain reaction) amplification of the relevant targeted regions with gene-specific primers and Sanger sequencing (BigDye Terminator v3.1; ABI 3500 capillary sequencer), with bidirectional confirmation and manual review of chromatograms.

### 2.10 Statistical analysis

All statistical analyses were performed in R (v4.4). Continuous variables are summarised as median and interquartile range (IQR) with range; categorical variables - as counts and percentages. Because subgroup sizes were small and distributions non-normal, only non-parametric methods were used. Genotype-group comparisons (founder allele carriers vs. non-carriers) used the two-sided Wilcoxon rank-sum test with exact p-values; effect sizes were quantified with Cliff’s delta, which is a nonparametric effect-size measure that tells how strongly two groups differ, especially when the data are not normally distributed (with the conventional negligible/small/medium/large interpretation). To control the false-discovery rate across the four functional endpoints (age at onset, BCVA, visual field diameter, spherical equivalent), p-values were adjusted by the Benjamini–Hochberg (BH) procedure. Associations between age and visual function were assessed with Spearman’s rank correlation. Two-sided p < 0.05 was considered nominally significant; genotype-phenotype comparisons are reported as exploratory.

## 3 Results

### 3.1 Cohort and diagnostic context

Within this cohort *EYS* accounted for 39 of 361 (10.8%) molecularly solved cases, placing *EYS* among the most frequent arRP causative genes in this population. There were 22 women and 17 men. Self-reported ethnicity (from passport data) was predominantly East-Slavic but also included Tatar, Mari, Chechen, Cherkess, Kazakh, Tajik, Ukrainian, and Belarusian individuals, reflecting the ethnic diversity of Eastern Europe and Northern Asia. Genetic ancestry (from WES) was concordant with the self-reported origin (Section 2.7). Median age at disease manifestation was 18 years, interquartile range (IQR) 15–19; range 9–25, and median age at examination was 29 years (IQR 24.5–37.5; range 15–66).

### 3.2 The *EYS* mutational spectrum

The *EYS* gene mutation spectrum consisted of 10 frameshift variants (29.4%), nine deletions/SVs (26.5%), seven nonsense variants (20.6%), five splice-region variants (14.7%), and three missense variants (8.8%) (Figure 1a). All reported variants are shown in Figure 1b depicting in which domain of the *EYS* gene the exact variant was located. Fifteen of the 34 variants (44.1%) were novel (Figure 1c) absent from gnomAD v4.1, ClinVar, HGMD, and published *EYS* literature at the time of analysis (Section 2.6). Eight of these novel variants were structural deletions, underscoring the high structural variant burden of the *EYS* locus and the value of dedicated copy number/SV analysis alongside exome sequencing (Zampaglione et al., 2020; de Bruijn et al., 2023; Fabian-Morales et al., 2024).

**FIGURE 1:**
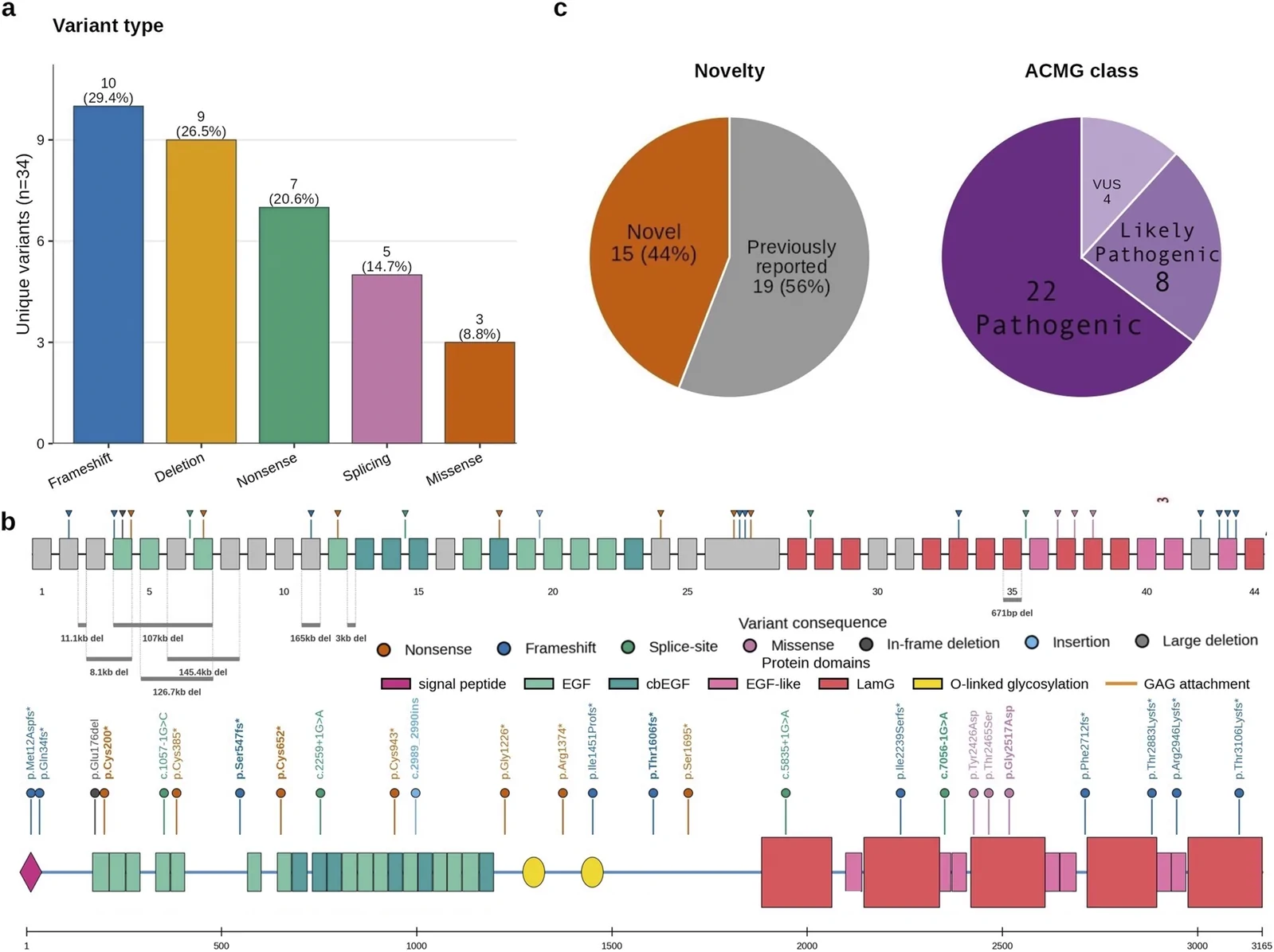
Spectrum and classification of *EYS* variants in the cohort. **(a)** Distribution of the 34 unique disease-causing *EYS* variants by molecular consequence: frameshift 10 (29.4%), deletion (structural/copy number) 9 (26.5%), nonsense 7 (20.6%), splicing 5 (14.7%), and missense 3 (8.8%). **(b)** Positions of the identified variants along the *EYS* protein domain architecture (laminin G, EGF-like and Laminin A/G domains). **(c)** Variant composition of the unique set: novelty (15 novel, 44%; 19 previously reported, 56%) and ACMG/AMP classification (22 pathogenic, 8 likely pathogenic, 4 VUS).

Across 39 patients we identified 34 distinct *EYS* variants: 22 variants were pathogenic and 8 were likely pathogenic; the remaining 4 were classified as VUS (Figure 1c). Each of 38 patients had two variants, one in each allele, and patient P2 carried three alleles: one known pathogenic variant in *EYS* c.1057-1G>C and two VUS variants in the second allele confirmed by segregation analysis: c.7394C>G and c.525_527delGGA. They were classified as “hot” VUS with potentially damaging effect. As the available evidence did not allow reliable discrimination between these two VUSs, their individual contributions to the disease phenotype remain uncertain. This resulted in total of 79 alleles: 30 pathogenic, 45 likely-pathogenic, and 4 VUS alleles, classified by ACMG/AMP criteria (Richards et al., 2015). VUSs are reported separately with explicit caveats (Section 2.5), they were not used to support a molecular diagnosis. Following the hot/cold VUS classification, we regard VUS that occur in trans with an established null allele and carry additional supporting criteria as “hot” (leaning pathogenic and warranting functional follow-up), and isolated VUS without such support as “cold.”

### 3.3 Two recurrent founder alleles and relatedness

Two alleles recurred at high frequency (Table 1). The nonsense variant c.1155T>A (p.Cys385*, exon 7) was carried by 16 of 39 patients (allele count 16/79; 20.3% of disease alleles), and the frameshift variant c.8648_8655del​CAT​GCA​GA (p.Thr2883Lysfs*4, exon 43) was carried by 15 of 39 patients (allele count 15/79; 19.0%). Together these two alleles accounted for 31 of 79 disease alleles (39.2%). Twenty patients carried at least one of the two recurrent alleles, and nine patients carried both.

To test whether this recurrence reflects shared ancestry rather than independent mutational events, we performed relatedness analysis on the exomes of the cohort using KING version 2.3.2 on a filtered, high quality SNP set. Across all 741 pairwise comparisons, only two pairs showed above-background kinship P2–P3 (a known mother–daughter pair) and P16–P32 (fourth degree, known as second cousins); all remaining 739 pairs were unrelated (Figure 2; Supplementary Data Sheet 1). This confirms that the recurrent alleles reflect population-level founder effects rather than close familial clustering within the cohort (Manichaikul et al., 2010).

**FIGURE 2:**
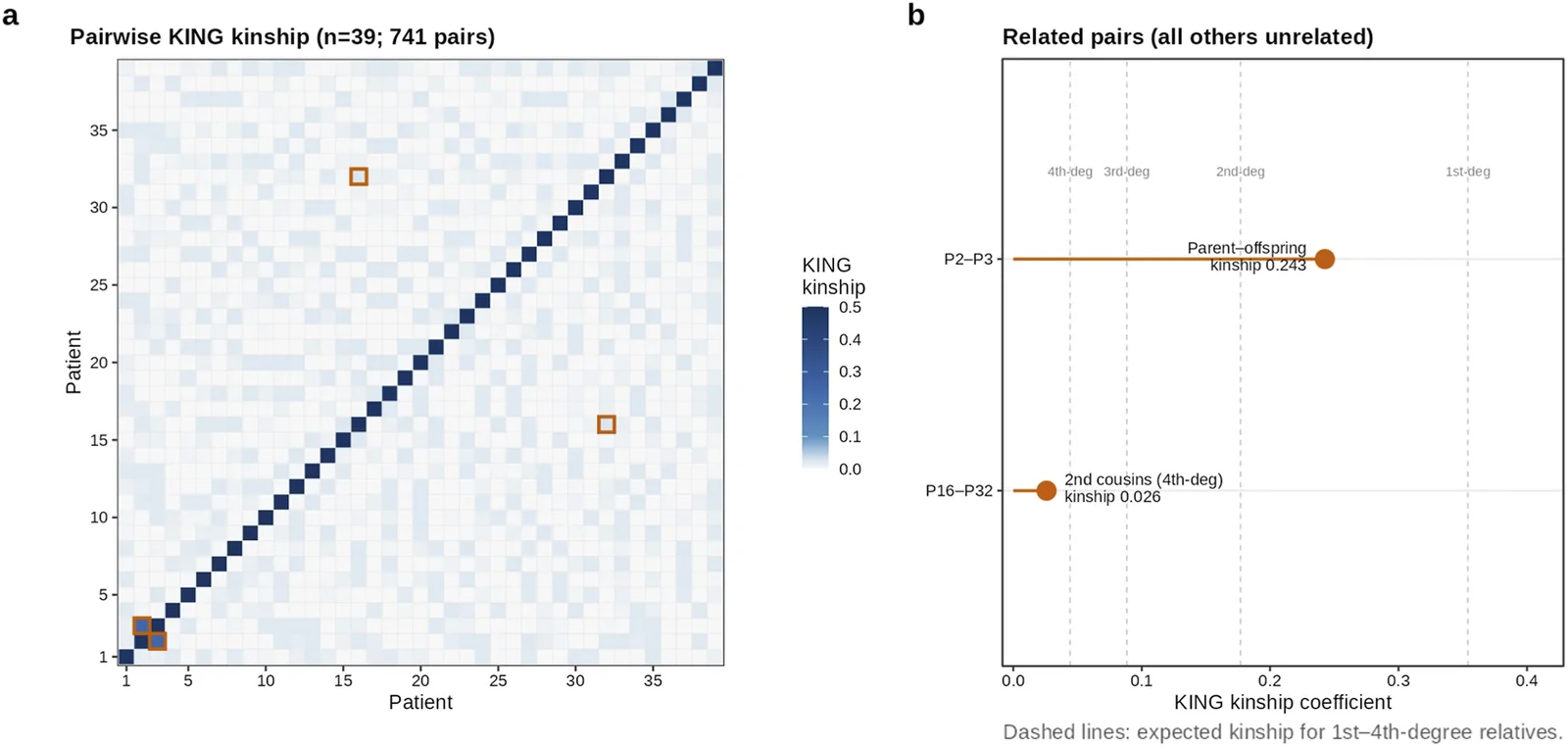
Genetic relatedness within the cohort. **(a)** Pairwise KING-software kinship coefficients for all 741 patient pairs (39 individuals); cells shaded by kinship (diagonal set to 0.5), with the two related pairs outlined. **(b)** Two related pairs against expected kinship with first- and fourth-degree relatives: P2–P3 (kinship 0.243, known mother–daughter pair) and P16–P32 (kinship 0.026, fourth degree; known second cousins). All remaining 739 pairs were unrelated.

### 3.4 Genotype–phenotype associations

Because the two founder alleles were very frequent in the cohort, we asked whether carrying a founder allele was associated with a distinct clinical phenotype. We compared key clinical parameters between founder allele carriers (patients carrying at least one of c.1155T>A or c.8648_8655del; n = 20) and non-carriers (n = 19). Both founder alleles are loss-of-function (a nonsense and a frameshift), so this contrast also serves as a genotype-based severity comparison within the cohort and is presented in Table 2.

**TABLE 2:** Genotype–phenotype comparison: founder allele carriers vs. non-carriers.

Endpoint	Founder-carrier (n = 20), median	Non-carrier (n = 19), median	Cliff’s delta δ	95% confidence interval	p (Mann-Whitney)	q (Benjamini–Hochberg)
Age at onset (y)	18	16	0.24	[-0.15, 0.57]	0.2	0.39
BCVA (logMAR)	0.16	0.16	0.06	[-0.29, 0.4]	0.75	0.75
Visual field size (degrees)	15	10	0.31	[-0.05, 0.6]	0.088	0.35
Spherical equivalent (Diopters)	−2.5	−1.5	−0.2	[-0.5, 0.16]	0.29	0.39

Two-sided Mann–Whitney test with exact p-values; Cliff’s delta (δ) effect size with 95% CI (confidence interval); q = Benjamini–Hochberg-adjusted p across the four endpoints. Founder-carriers n = 20; non-carriers n = 19. No endpoint was significant after correction was observed (all q ≥ 0.35). logMAR: higher = worse acuity.

To assess potential differences between the two patient groups across the four clinical endpoints, we used non-parametric two-sided Wilcoxon rank-sum tests. This rank-based approach was chosen to ensure robust comparisons without assuming a normal distribution, preventing skewed data or extreme outliers from distorting the results. Statistically significant difference was not observed in these founder allele carriers (n = 20) and non-carriers (n = 19) groups.

To complement hypothesis testing with a direct measure of physical magnitude, we determined Cliff’s delta (δ) effect sizes for each outcome. This non-parametric coefficient ranges from −1 to +1 and quantifies the degree of overlap between the two group distributions, offering insight into any difference independent of sample size. Also, evaluating four distinct clinical endpoints simultaneously increases the risk of false-positive results. To address this, we applied the Benjamini–Hochberg procedure to adjust the p-values (q), effectively controlling the false discovery rate across all secondary comparisons.

Although age at onset was slightly higher in the carrier group, suggesting that founder alleles may be less severe, our study lacked sufficient statistical power to demonstrate statistical significance. Median of manifestation age was 18 vs. 16 years: p = 0.20, δ = 0.24; q = 0.39. Also, we observed that carriers exhibited a slightly higher refractive error, with mean spherical equivalent of −2.5 D compared to −1.5 D in non-carriers (p = 0.29, δ = −0.20; q = 0.39). However, BCVA was the same with mean 0.16 vs. 0.16 logMAR (p = 0.75, δ = 0.06; q = 0.75). Supporting hypothesis of founder alleles being less severe visual field diameter in founder allele carriers was slightly wider 15° degrees compared to non-carriers (10° degrees) with p = 0.088, δ = 0.31; q = 0.35. This comparison is exploratory and, given the modest subgroup sizes, is not powered to exclude small effects.

Clinical characteristics of the cohort in general is presented in Table 3 with details of carriers vs. non-carriers comparison in Figures 3a–d. Two patients who carried a missense allele in trans with a predicted loss-of-function allele: P4 (c.1155T>A nonsense + c.7276T>G missense) and P37 (c.1155T>A nonsense + c.7550G>A missense) were observed to present with less severe phenotype. Two patients carried the c.32dupT (p.Met12Aspfs*14) allele in the heterozygous state (P1 and P7) with a defined second null allele, patient P1 with c.4350_4356del (p.Ile1451Profs*3) and patient P7 with c.4815_4816delCAinsATAG (p.Thr1606fs) (Supplementary Table 1). We observed more cone involvement in these two patients primarily because severe, early truncating null allele abolishes protein function, and accelerates cone photoreceptor degeneration alongside typical rod loss.

**TABLE 3:** Cohort clinical characteristics (n = 39).

Variable	Value
N	39
Female	22
Male	17
Age at onset (years)	median 18 (IQR 15–19; range 9–25; n = 39)
Age at exam (years)	median 29 (IQR 24.5–37.5; range 15–66; n = 39)
BCVA (decimal)	median 0.70 (IQR 0.40–0.80; range 0.15–1.00; n = 39)
BCVA (logMAR)	median 0.16 (IQR 0.10–0.41; range −0.00-0.85; n = 39)
Visual field diameter (degrees)	median 15.00 (IQR 10.00–17.50; range 5.00–20.00; n = 39)
Spherical equivalent (Diopters)	median −2.50 (IQR -2.50--0.75; range −7.50-0.50; n = 39)
Macular complication n(%)	12 (31%)
Cataract/pseudophakia n(%)	13 (33%)

Values are median (IQR - interquartile range; range) for continuous variables and n (%) for categorical variables. BCVA, best corrected visual acuity; logMAR, logarithm of the minimum angle of resolution; D, diopters.

**FIGURE 3:**
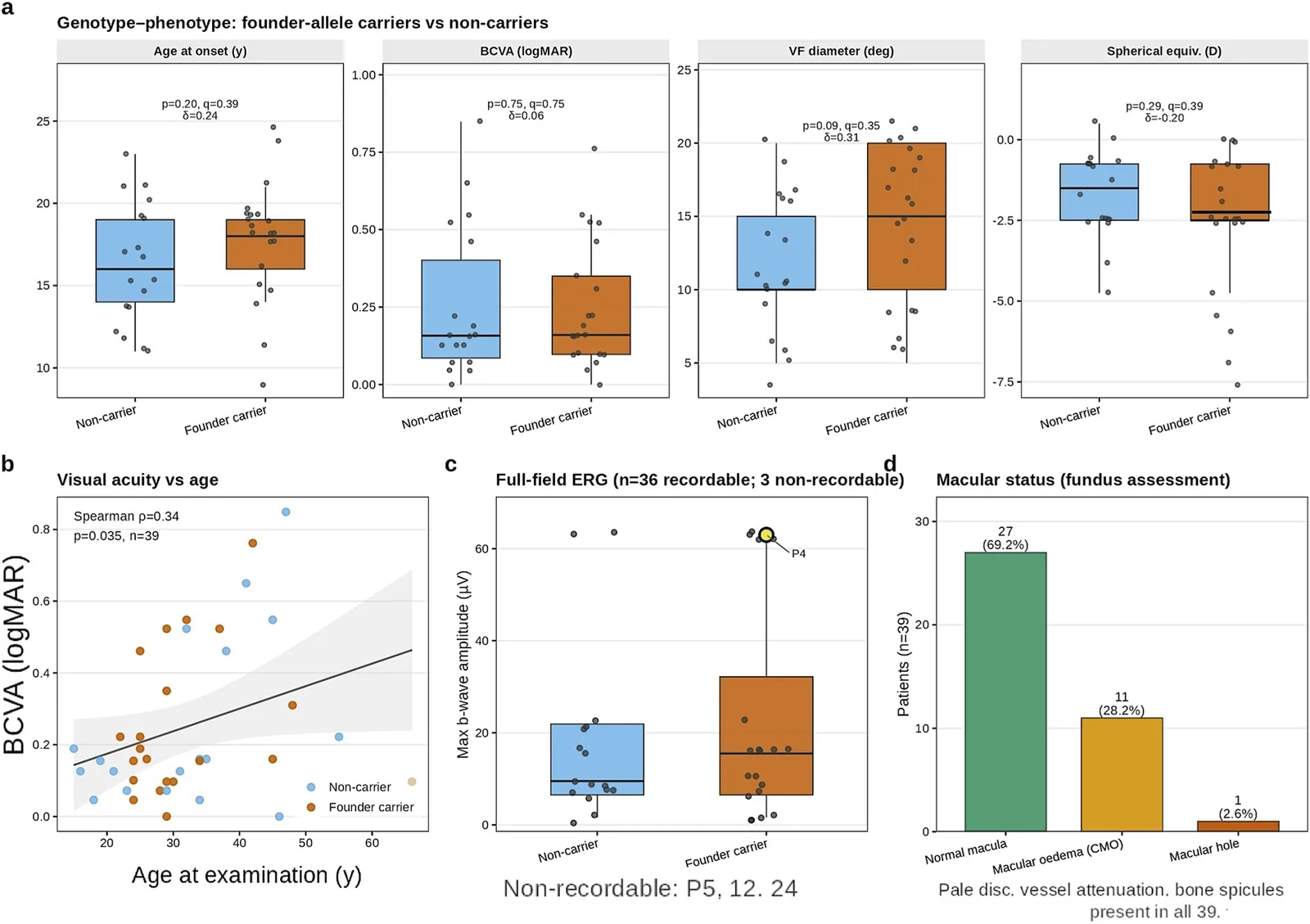
Genotype–phenotype relationships and retinal function. **(a)** Key clinical endpoints in founder allele carriers (n = 20) versus non-carriers (n = 19): age at onset (years), BCVA (logMAR), visual field size (degrees), and spherical-equivalent refraction (diopters). Boxes show median and interquartile range; points are individual patients; two-sided Wilcoxon p, BH-adjusted q, and Cliff’s delta (δ) are annotated. **(b)** Age at examination versus BCVA (logMAR) across all 39 patients, with linear trend and 95% band (Spearman rank correlation coefficient = 0.34, p = 0.035). **(c)** Full-field ERG maximal (dark-adapted) b-wave amplitude per patient, colored by founder-carrier status; 36 recordable (1.6–63.0 µV), 3 non-recordable (P5, P12, P24). **(d)** Macula status on ophthalmoscopic assessment: normal 27 (69.2%), cystoid macular oedema 11 (28.2%), macular hole 1 (2.6%).

Considering the cohort as a whole, worse visual acuity was associated with older age at examination (Spearman rank correlation coefficient ρ = 0.34, p = 0.035; Figure 3b). Visual field diameter expectedly showed correlation with age (Spearman ρ = 0.013, p = 0.045), and age at onset was not correlated with visual acuity (Spearman ρ = −0.12, p = 0.47). These findings are consistent with the slowly progressive rod–cone dystrophy expected for *EYS*-associated disease.

### 3.5 Retinal imaging and electrophysiology

Fundus examination and full-field ERG were consistent with a progressive rod–cone dystrophy. Pale optic disc, attenuated retinal vessels, and bone-spicule pigmentation were universal (present in all 39 patients). Macular complications on fundus/ophthalmoscopic assessment, cystoid macular oedema and, in one patient, a macular hole (Figure 3d), were seen in 12 of 39 patients (31%), and cataract or pseudophakia was present in 13 of 39 (33%) (Table 3). Consistent with classic RP, rod function was affected earliest with later cone involvement, and central vision was comparatively preserved (Marques et al., 2023; Martins et al., 2024; Mucciolo et al., 2018).

Full-field ERG was available for the cohort (Figure 3c). Dark-adapted (maximal) responses were severely reduced across patients, in keeping with a rod–cone dystrophy: 36 patients had recordable maximal b-wave amplitudes (range 1.6–63.0 µV) and three had non-recordable ERG (P5, P12, P24). One founder-carrier retaining comparatively preserved retinal function (P4; a-wave 43 μV, b-wave 63 µV). These observations are descriptive; no formal statistical testing of ERG amplitudes was performed.

## 4 Discussion

This study provides the first systematic description of the *EYS* mutational and clinical spectrum in an Eastern European and Northern Asian cohort. In 39 patients we identified 34 distinct *EYS* variants, 15 of them novel (including eight novel structural deletions), and defined two recurrent alleles: c.1155T>A (p.Cys385*) and c.8648_8655del (p.Thr2883Lysfs*4), that together account for 39.2% of disease alleles and are consistent with a shared founder origin. These findings expand the known allelic diversity of *EYS* and identify population-specific targets for cost-effective first-tier screening in this region.

### 4.1 A high structural variant burden

Eight of the 15 novel variants were structural deletions, and large deletions/SVs constituted roughly a quarter of the entire variant spectrum. This high SV burden is characteristic of the *EYS* locus, which spans nearly 2 Mb, and reinforces that comprehensive *EYS* diagnostics require dedicated copy number and structural variant analysis (exome-based copy number variations (CNV) calling with MLPA confirmation) in addition to small-variant detection. Diagnostic pipelines that rely on small-variant calling alone will systematically under-ascertain *EYS* disease causality (de Bruijn et al., 2023; Fabian-Morales et al., 2024; Zeuli et al., 2024).

### 4.2 Founder alleles in *EYS* across populations

The distribution and frequency of *EYS* mutations vary substantially among ethnic groups. In Chinese (Sun et al., 2020; Gao et al., 2022), Japanese (Yang et al., 2020; Arai et al., 2015; Iwanami et al., 2019; Koyanagi et al., 2019), Korean (Park et al., 2025), Taiwanese (Chen et al., 2021), and Singapore (Teo et al., 2021) populations, pathogenic variants: c.4957dupA (p.Ser1653Lysfs*2) and c.8805C>A (p.Tyr2935*) are the most prevalent, accounting for a significant proportion of *EYS*-related RP cases. In India the most frequent variant in *EYS* is 2259+1G>A (Di et al., 2016) and in Iberia and Iran a ∼60.1 kb deletion of exons 13-14 c.(2023+1_2024-1)_(2259+1_2260-1)del is the most frequent variant (Heutinck et al., 2025). In European populations, recurrent variants including c.9253_9254delGA (p.Glu3085Ilefs*4) and c.6416G>A (p.Cys2139Tyr) are more commonly detected as we can see from the Dutch (Littink et al., 2010), Spanish (Barragán et al., 2010), Italian (Zeuli et al., 2024), Portuguese (Marques et al., 2023) and Swedish (Westin et al., 2021) reports. In the USA (Zampaglione et al., 2020), Mexico (Fabian-Morales et al., 2024) and Brazil (Martins et al., 2024), there is a greater diversity of RP causing *EYS* mutations. Data is scarce for Arab (Jaffal et al., 2021) and African (Habibi et al., 2016) countries. We examined whether the recurrent Russian alleles could share reported founder origins. Genetically inferred ancestry in our cohort was East and North Slavic and Central-Asian. Taken together, the Russian recurrent alleles represent independent, population-specific founder events (Figure 4).

**FIGURE 4:**
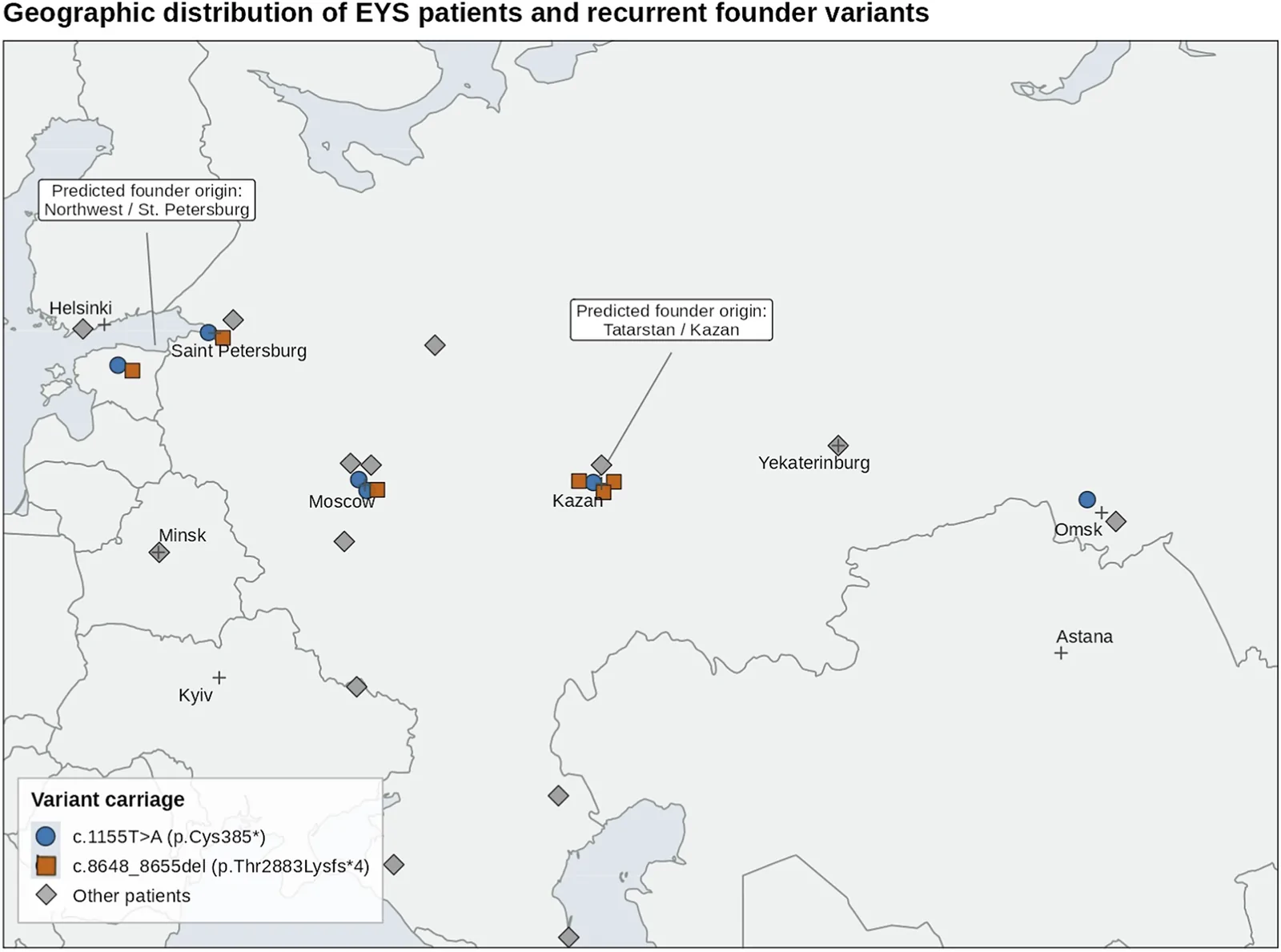
Geographic distribution of patients and recurrent founder variants. Approximate residence localities of the 39 patients across Eastern Europe and Northern Asia on a political base map. Blue circles mark carriers of c.1155T>A (p.Cys385*) and orange squares mark carriers of c.8648_8655del (p.Thr2883Lysfs*4); grey diamonds mark the remaining patients. Carriers of both founder alleles concentrate in the Northwest (Saint Petersburg region) and in Tatarstan (Kazan region), supporting two independent regional founder origins; the Moscow region shows a mixture of variants. Marker positions are approximate, and where several patients share locality markers are offset for visibility.

### 4.3 Genotype–phenotype observations

The cohort presented a homogeneous, classic rod–cone dystrophy with relatively preserved central vision, in keeping with previous *EYS* published data (Marques et al., 2025). A formal comparison of founder allele carriers versus non-carriers showed no significant differences across the functional endpoints examined (all q ≥ 0.35), and the cohort is not powered to detect modest genotype effects. We nonetheless note, as a descriptive observation, that the two patients carrying a missense allele in trans with a null allele (P4 and P37) tended towards a milder course later onset, wider residual fields, more myopic refraction, and, in P4, a comparatively preserved maximal ERG b-wave consistent with the possibility that residual *EYS* function attenuates disease. This hypothesis warrants testing in larger, adequately powered cohorts (Soares et al., 2023).

Beyond the classic presentation, atypical *EYS* phenotypes including comparatively preserved or regionally atypical retinal findings are increasingly recognized, like macular-predominant degeneration, or unusually late/mild disease, with more high myopia-associated refraction, sector RP or regional retinal degeneration, unusual ERG pattern, disproportionate cone dysfunction or relatively preserved rod responses compared with classic *EYS*-RP (Pierrache et al., 2019). This atypical prevalence and mechanistic basis have been reviewed recently (Barthelemy et al., 2026). Systematic recognition of atypical presentations is important to avoid clinical under-diagnosis of *EYS*-associated disease.

### 4.4 Gene-based therapy

Establishing recurrent founder alleles has direct translational relevance. The predominance of a small number of alleles makes *EYS* an attractive candidate for mutation-directed approaches (for example, base or prime editing, or antisense oligonucleotide strategies) targeting the specific recurrent variants prevalent in this population. However, gene-supplementation for *EYS* remains constrained by the size of its coding sequence (∼10 kb), which exceeds the packaging capacity of a single adeno-associated virus vector, motivating dual-vector, non-viral, or gene-editing strategies. The two recurrent Russian alleles: a nonsense and a small deletion are in principle tractable to editing-based correction, and defining their frequency is a prerequisite for prioritising such approaches regionally.

### 4.5 Unsolved cases and non-coding variants

Comprehensive evaluation of the *EYS* locus requires interrogation beyond the coding exons, because a substantial number of possibly pathogenic alleles are deep-intronic or structural (Huang et al., 2025; Hayman et al., 2025). Within the cohort of 500 people with RP from the described program, six unsolved patients carried a single heterozygous pathogenic/likely-pathogenic *EYS* allele without an identified second variant: 1) c.8648_8655del​CAT​GCA​GA (p.Thr2883Lysfs*4), 2) c.9317_9336del (p.Thr3106LysfsTer13) 3) c.5053T>G (p.Ser1685Ala) 4) 93,5 Kb deletion of exons 4 – 7 with coordinates (hg38) chr6:65389883-65483419 5) c.6714del (p.Ile2239SerfsTer) and 6) c.3489T>A (p.Asn1163Lys). In these unsolved cases a second, non-coding or structural allele is likely and would require genome sequencing, long-read sequencing, and functional assays to resolve. If these unsolved cases will be confirmed with the second mutation in the second allele of *EYS* gene, that can increase diagnostic yield of *EYS*-causative RP in this cohort to up to 12.46%.

### 4.6 Strengths and limitations

Strengths of this study include a well-phenotyped, genetically confirmed cohort; comprehensive small-variant and structural variant analysis with MLPA confirmation; formal statistical treatment of genotype–phenotype relationships with acknowledgement of power in founder allele carriers versus non-carriers; and the first definition of *EYS* founder alleles in this population. Limitations include the cross-sectional design, which precludes direct measurement of progression rates and the small size of the subgroups, which limits genotype–phenotype inference.

## 5 Conclusion

In the first systematic analysis of *EYS*-associated RP in an Eastern European and Northern Asian cohort, we expand the *EYS* mutational spectrum with 15 novel variants (eight novel structural deletions) and define two population-specific founder alleles, c.1155T>A (p.Cys385*) and c.8648_8655del (p.Thr2883Lysfs*4), that together account for 39% of disease alleles. The cohort shows a homogeneous rod–cone dystrophy; founder allele carriers and non-carriers did not differ significantly in the examined clinical parameters. These findings support targeted first-tier screening for the recurrent alleles in this region and provide a genotype-phenotype foundation for future gene-based therapeutic development.

## Data Availability

The original contributions presented in the study are included in the article/Supplementary Material, further inquiries can be directed to the corresponding author.
